# Bryonia

**Published:** 1892-06-04

**Authors:** 


### THERAPEUTICS.

### BRYONIA.

The white bryony, *Bryonia alba*, is a plant which is sufficiently well known to all lovers of field flowers and shrubs, but as a medicinal agent is but little used in Europe. It is a perennial climbling plant, growing plentifully, and generally found in English hedges and thickets. The black berries are sometimes eaten by children, and many cases of bryony poisoning have occurred. Another species, Bryonia dioica, having red berries, is also used medicinally. A tincture prepared from the roots of these two species has been officinal in the U.S. Pharmacopœia for several years, and its value is more widely recognised across the water than it has been hitherto in this country.

The U.S. Dispensatory states that as kept in the shops, the root is in circular transverse slices, decidedly yellowish grey, and longitudinally wrinkled, internally of a whitish colour, becoming darker by age, concentrically striated, light, brittle, and readily pulverisable, yielding a whitish powder. Besides a peculiar bitter principle, called bryonin, the root contains starch in considerable proportion, gum, resin, sugar, a concrete oil, albumen, and various salts. It yields its active properties to water. Bryonin may be obtained by mixing the powdered root with one-sixth its weight of purified animal charcoal, in fine powder, putting the mixture into a percolator, already containing a quantity of animal charcoal equal to that mixed with bryony, and then percolating successively with alcohol, diluted alcohol, and sufficient water to displace the alcoholic liquid. The tincture thus obtained yields the bryonin by spontaneous evaporation. This is extremely bitter, soluble in water and alcohol, insoluble in ether, unaltered by the alkalies, and dissolved by sulphuric acid, with the production of a blue colour. It purges actively in the dose of two grains. And, as regards its medical properties, this authority states that bryony is an active hydragogue cathartic, in large doses sometimes emetic, and disposed, if too largely administered, to occasion inflammation of the alimentary mucous membrane. It appears also to have direct relations with the nervous system, and it has, in a number of cases, produced serious or even fatal poisoning. Vomiting and purging have been commonly present, but in some cases there has been no diarrhœa. Giddiness, delirium, and dilated pupils, coupled with a fall of the bodily temperature, imperceptible pulse, cold perspiration, and other manifestations of collapse are the usual symptoms.*

Dr. Phillips and Dr. Lauder Brunton have recommended that bryonia, in frequently repeated small doses, should follow the use of aconite in the earliest stages of pleurisy. Bryonia seems to have a definite action on serous membranes.

Dr. A. Storrs † has used the drug for several years in pleurisy, and has the highest opinion of its use in this disease, never having had effusion except in one case complicated with other complaints, and in which it seems to have been administered when effusion had already occurred. He has also used it in bronchitis, and in pleuro-pneumonia with very good results. Unless the patients have been ill for several days before being seen, he gives them tincture of aconite ♏vi., aq. chloroform ʒii., aq. ad. ℥iii., one tablespoonful every hour. To the second bottle he adds tincture bryoniæ ♏xxiv., and gives it every two hours. In bronchitis or pneumonia he sometimes adds three doses of ipecacuhana wine to the first bottle (about ♏xxx), and in most cases, when he has used bryonia, after two bottles have been taken, the temperature is either normal or about 100 degrees. Bryonia, he states, is, in large doses, a hydragogue cathartic; in small doses it has a specific action on the pleura, and, he thinks, on the lung also. It is antipyretic, acts on the liver, and is a good remedy in rheumatic affections. Mr. H. Rainsford has recently reported a case of pneumonia which he treated with very small doses of tincture of bryonia. A young man, aged thirty, had been feeling ill one day, and when he was first seen he had a temperature of 102 degrees. The temperature gradually rose, and on the fourth day of illness had reached 103 degrees. He had then profuse rusty sputum and marked crepitation over the right base. On this, the fourth day, the patient was put on half-ounce doses of a mixture containing tincture bryoniæ ♏xx., aqua dist. ad. ℥vi. On the sixth day the lung was solid, no air entering the lower lobe on the right side. On the seventh day crisis occurred, the temperature falling to normal. It appeared to Mr. Rainsford that the drug materially modified the course of the disease, and prevented it from assuming its usual severity.

It would seem that bryonia exerts its beneficial action in the early stages of inflammation of the pericardium and peritoneum, as well as of the pleura.

* *U.S. Dipensat.*, p. 308.

† *British Medical Journal*, May 7th, 1892; and *Ibid*, April 9th, 1892.

